# A Novel Battery to Assess “Cool” and “Hot” Executive Functions: Sensitivity to Age Differences in Middle Childhood

**DOI:** 10.3390/brainsci14080755

**Published:** 2024-07-27

**Authors:** Laura Fernández-García, Jessica Phillips-Silver, María Teresa Daza González

**Affiliations:** 1 Department of Psychology, University of Almería, 04120 Almería, Spain; lfg403@ual.es; 2 CIBIS Research Center, University of Almería, 04120 Almería, Spain; 3 Growing Brains, Washington, DC 20394, USA; jessicaphillipssilver@gmail.com

**Keywords:** executive functions, neurodevelopment, middle childhood, theory of mind, cognitive control

## Abstract

The main goal of the current work was to assess the age sensitivity of a novel battery of cool and hot Executive Function (EF) tasks developed for the middle childhood period: the Executive Brain Battery (EBB). To this end, we carried out a first study in which the EBB was administered to six age groups ranging from 6 to 11. Additionally, in a second study, we compared children at the end of middle childhood (age 11 years) and adult performance in the EBB. Results showed that tasks included in the EBB were suitable for all age groups, with more age-related changes being found in cool than hot EF tasks. Moreover, at the end of middle childhood, children reach an adult-like performance in most of these cool and hot tasks. The present findings extend previous research suggesting that cool and hot EFs exhibit different patterns of age-related growth in middle childhood. Additionally, the EEB could become a useful tool for research on EFs during middle childhood that could be adapted for a wide range of populations.

## 1. Introduction

During the initial phase of middle childhood, typically between 6 and 11 years old, children begin formal schooling, entering an environment characterized by increased cognitive, emotional, and social demands. This period necessitates the development of Executive Functions (EFs) to enable children to sustain attention and motivation, navigate distractions during classes, achieve academic goals, and effectively regulate emotions. Adapting behavior and engaging with peers also rely on well-developed EFs. Consequently, it is unsurprising that numerous studies have identified strong associations between EFs and school readiness, academic performance, and social behavior [1,2,3].

Traditionally, research on EFs in adults and children has focused almost exclusively on the cognitive components. However, two key developments prompted researchers to recognize the importance of analyzing EFs within the emotional and social context. First, Damasio et al. [4] formulated the somatic marker hypothesis based on patients with lesions in the ventromedial prefrontal and orbitofrontal cortex. These patients showed emotional alterations, such as difficulty in decision making, but not in other cognitive components traditionally associated with EFs and prefrontal cortex lesions. Another development described by Mischel et al. [5] was the ability to delay gratification, also related with the ventromedial prefrontal cortex, highlighting the emotional aspects of EFs. Thus, in recent years, a holistic model of EFs has gained prominence, distinguishing between cognitive (or cool) EFs and socio-emotional (or hot) EFs [6,7,8,9,10,11].

According to this distinction, cool EFs include the cognitive processes necessary for solving decontextualized problems, typically activated without any affective, motivational, or social interaction component. These include *updating of working memory* (WM), *inhibitory control* (including the ability to inhibit an automated response and suppress distractor interference), and *set shifting*. In contrast, hot EFs come into play during social and affective situations that evoke emotions, motivation, and tension between immediate gratification and greater long-term rewards. The few previous studies exploring the more socio-emotional components of EFs include *decision making under uncertainty*, *delay of gratification*, and *affective reversal learning* (or the ability to adjust our behavior when there is a change in previously learned contingency relationships). Another component within hot EFs that some authors have included is the *theory of mind* (*ToM*) [12]. 

In the cool and hot model of EFs, it is important to emphasize that while a distinction is made between the cool and hot components, they are integral parts of a coordinated system relying on the same underlying processes [9,10]. Neuroimaging studies, for instance, have observed the recruitment of specific regions, such as the right ventrolateral prefrontal cortex, in both emotion-generating situations and those not involving affective processing [10]. Additionally, from a behavioral standpoint, limited studies incorporating this cool and hot EF model in middle childhood have noted a close relationship between the cool and hot components. For instance, some authors have identified significant correlations between visuospatial working memory and *decision making under uncertainty* among individuals aged 6 to 11 [13]. Hoyo et al. [14] reported notable correlations between visuospatial working memory and task switching (another subtype of cognitive flexibility) with tasks assessing *ToM*, particularly when an understanding of false beliefs was required. 

In a recent systematic review, Fernández García et al. [12] highlighted a substantial increase in studies on age-related changes in EFs during middle childhood over the past decade. However, drawing conclusions about the patterns of age-related growth in cool and hot EFs during this phase is challenging, based on the results of these studies. This challenge could be attributed, in part, to the following limitations. 

First, in some of these studies, the EF tasks are based exclusively on models of brain damage in adults, without adapting these measures to children’s cognitive development according to their age [15]. This complicates the analysis of age-related changes in cool and hot EFs and hinders progress in detecting and understanding developmental alterations. Second, complex EF tasks often involve implementing several partially overlapping cool and hot EFs [16]. Miyake et al. [17] termed this limitation the task impurity problem. For example, the “Wisconsin Card Sorting Test” (WCST; [18]) requires participants to infer the classification rule on each trial (sorting cards based on color, shape, or number of stimuli). Participants must maintain working memory for previous rules, flexibly change the rule with negative feedback, and suppress interference from other stimulus dimensions. Despite its popularity, on numerous occasions, the WCST has been unable to distinguish between patients with frontal and non-frontal brain injuries [19]. 

Moreover, very few previous studies (longitudinal or cross-sectional) have explored both cool and hot EFs across the entire age range of middle childhood (from 6 to 11 years of age). Additionally, the assessment tools and measures used in previous studies tend to vary considerably (for a review, see [12,20]). Likewise, very few previous studies have compared children in middle childhood and adults in terms of performance on the same tasks to assess some cool and hot EFs. In this regard, it would be useful to have a battery of tasks that assess, in a single session, both cool and hot EFs across the entire age range of middle childhood (ages 6–11), since some researchers have tried to create different batteries of performance-based tasks to provide a set of measures for gaining deeper insights into EFs during middle childhood (see Table S1 in Supplemental Materials). However, most of these task batteries were not based on a theoretical model of EF. Consequently, given the cool and hot EF model presented earlier, existing batteries may be limited in terms of assessing EFs. As can be seen in Table S1 of the Supplemental Materials, some of these batteries only allow evaluating certain components of the cool EFs (e.g., “Delis-Kaplan Executive Function System”—K-DESF [21]; “NIH Toolbox Cognition Battery”—[22]; “Neuropsychological Assessment of Executive Functions Battery for Children”—ENFEN [23]; or the “Children’s Neuropsychological Assessment Battery”—ENI [24]. Additionally, some batteries do not include tasks to assess all the cool and hot components of EFs. For example, only decision making in a risk situation is evaluated in the Cambridge Neuropsychological Test Automated Battery” (CANTAB; [25]), while in the “Neuropsychological Battery of Executive Functions and Frontal Lobes” (BANFE; [26]), only *decision making in situations of uncertainty* is evaluated. Moreover, the “Child Neuropsychological Battery” (NEPSY-II, [27]) includes a task that measures *ToM* ability, but the authors of the battery did not consider this ability as another EF.

### Current Study

Given the considerations mentioned above, for the present work, a new battery of computerized tasks, named the *Executive Brain Battery* (EBB), was designed for the specific assessment of both cool and hot EFs. The EBB is tailored for children aged 6 to 11–12 years. In developing the EBB, we aimed to evaluate the cool and hot EFs outlined in the holistic model mentioned earlier. Consequently, we included EF tasks adapted for children in the specified age range, allowing us to measure cool EFs: (1) *updating of working memory*, (2) *inhibitory control (covering both inhibition of automated responses and interference suppression)*, and (3) *set shifting*. We also measured hot EFs: (4) *decision making under uncertainty*, (5) *delay of gratification*, (6) *affective reversal learning*, and (7) *theory of mind*. 

Furthermore, considering the results reported in the systematic review by Fernández García et al. [12], the EF tasks included in our EBB were designed according to the following criteria: (1) The tasks had to be based on the paradigms most used in research on cool and hot EFs in children between 6 and 12 years of age. (2) Tasks must have previously demonstrated some sensitivity to the changes in EFs during this developmental period. (3) The instructions and execution of each task must involve minimal verbal content to cater to children with a different first language or those with language delays (for example, deaf children, children with Specific Language Disorders, or dyslexia, among others). For instance, this explains why the EEB did not include a Stroop task to assess inhibitory control despite its frequent use in developmental research [12]. Since the Stroop task relies on automated word reading to obtain a measure of inhibitory control, our EBB included a task based on the “Flanker paradigm” [28], which involves suppressing the interference of distractors without the use of verbal stimuli. 

In this regard, the main aim of the present work was to explore the viability of cool and hot EF tasks included in the EBB for children aged 6–11 years and analyze their sensitivity to age-related changes during middle childhood. To do this, we report the results of two studies with the EBB. 

In Section 2, cool and hot EF tasks from the EBB were administered to six age groups ranging from 6 to 11. Thus, we expect that most of the tasks included in the EBB will be practicable for most of the children in this study, given that they were designed based on the paradigms traditionally used to evaluate EFs in this age range. Furthermore, we expect the tasks to be sensitive to age-related changes in most cool and hot EFs.

One of the limitations in the evaluation of EF in children is the use of tasks designed for adult patients with brain damage. These tasks are not usually designed within a motivating context for children that helps them activate all their cognitive resources during the task (for example, pressing the space bar when a green circle appears on a white screen). In Section 3, a group of adults was included with the aim of examining whether the tasks could be used in future comparative studies to explore age-related changes in EFs. To do this, the performance of children at the end of middle childhood (11-year-old children) and adults on the EBB was directly compared. We expected not to find any ceiling or floor effects, given that the tasks are based on experimental paradigms traditionally used in both children and adults. However, the tasks included in the EBB are designed in a more friendly and motivating context for the child population.

## 2. Study 1

The aim of the current study was three-fold: (1) design a new battery of computerized tasks for the specific assessment of both cool and hot EFs, which could be suitable for both 6 and 11–12 year old children: the *Executive Brain Battery*; (2) assess the feasibility of tasks included in the EBB for evaluating cool and hot EFs in children aged between 6 and 11; and (3) analyze the sensitivity of the EBB to age-related changes in cool and hot EFs during middle childhood. 

Given that the EBB was developed based on the findings from the systematic review by Fernández García et al. [12], we anticipated that both cool and hot EF tasks would be feasible for most children across all age groups (6–11 years). Moreover, considering the limited prior research on the development of cool and hot EFs in middle childhood, we expected to observe age-related improvements in performance on both cool and hot EF tasks, particularly in the cool EF tasks.

### 2.1. Materials and Methods

#### 2.1.1. Participants 

A total of 202 children aged between 6 and 11 years (M_age_ = 8.50, SD = 1.47, 50% female) participated in this study, recruited from a public educational center. Seven children were excluded from the analysis because they did not meet the inclusion criteria, which specified that participants should not present any neurological or psychiatric disorder. 

The final sample comprised 195 children (M_age_ = 8.63, SD = 1.52, 50.8% female) divided into six age groups. Information regarding the age, gender, and educational level of the parents can be found in Table 1. Regarding educational level, both the fathers and mothers’ years of study were categorized into four levels: low (6 to 8 years and ESO Compulsory Secondary Education), medium (High school and Higher grade), high (University studies), and unknown (parents who did not indicate their educational level). In cases where both parents reported their educational level, the highest level from either parent was considered for analysis. The chi-square test revealed no differences among the six age groups based on parental educational level (X^2^(15) = 22.451, *p* = 0.097) or gender (X^2^(5) = 8.188, *p* = 0.146).

#### 2.1.2. Measures: Executive Brain Battery (EBB)

The EBB consisted of 8 computerized tasks based on the experimental paradigms traditionally used to evaluate the hot and cool executive components (see Table 2). Previous studies have emphasized that children exhibit increased engagement in evaluation tasks when presented with a narrative and clear feedback on their performance [29]. Thus, the EBB tasks were framed as a series of games where participants assist the main characters in completing a mission. By contextualizing the tasks in this manner, we aimed to sustain motivation and cooperation among participants throughout the evaluation. 

All tasks were programmed with E-prime software [30], which controlled the presentation of the stimuli and the collection of the participants’ responses. Below, we present a brief description of each task. 

**Table 2:** Tasks included in Executive Brain Battery (EBB).

Task Name	EF Measured	Paradigm	Studies with Children
***The Colored Owls***	Updating of working memory	2-back	Ludyga et al. [31]
***The Zoo Game***	Inhibition of automated responses	Go/No-Go	Lamm et al. [32,33]
***Catch the Frog***	Interference suppression	Flanker	Traverso et al. [34,35]
***Find the Way***	Set shifting	Dimensional Change Card Sort test	Zelazo et al. [36]
***The Hungry Donkey***	Decision making under uncertainty	The Hungry Donkey task	Crone and van der Molen [37,38]
***Planes and Rewards***	Delay of gratification	Delay discounting	de Water et al. [39]
***The Mechanic’s Game***	Affective reversal learning	Probabilistic reversal learning	Van Duijvenvoorde et al. [40]
***Playing with Jack***	Theory of mind (understanding false beliefs)	Sally and Anne task	Salhi et al. [41]

**Figure 1:**
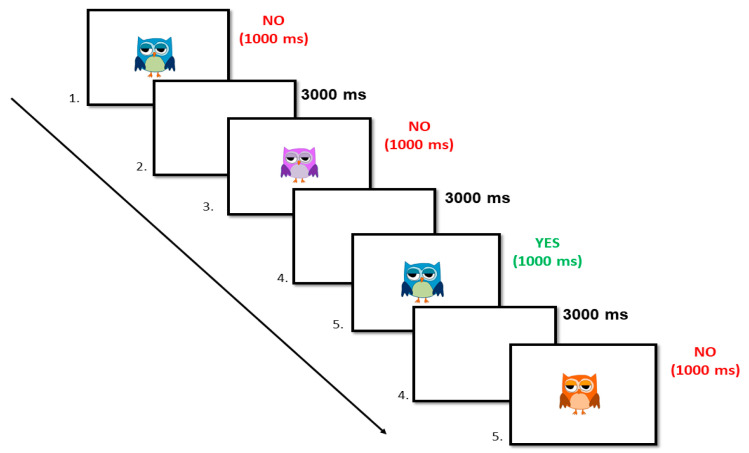
An illustrative example of a Target and No-Target trial sequence and stimulus presentation times.

**Figure 2:**
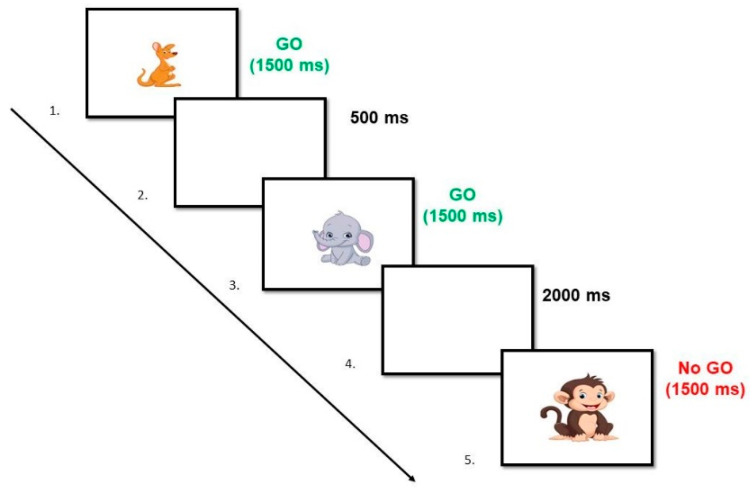
Example of a hypothetical sequence of “GO” and “No-GO” trials of the “The Zoo Game” task.

**Figure 3:**
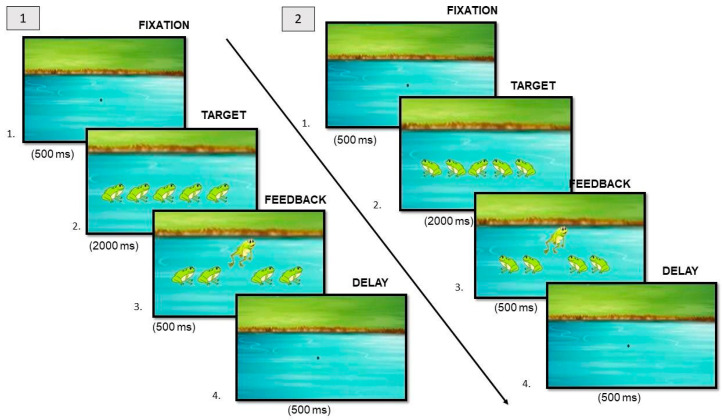
Example of congruent (**1**) and incongruent (**2**) trials of the “Catch the frog” task in which the participant gave the correct answer.

**Figure 4:**
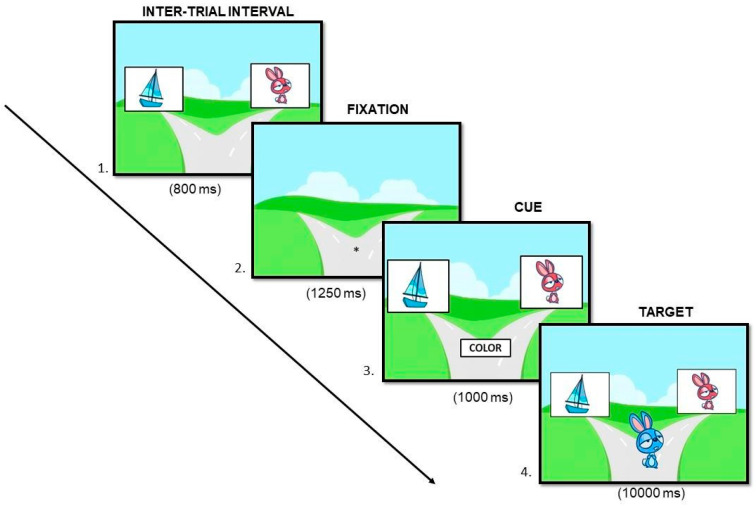
The trial sequence in which the participant has to classify the stimulus according to the color dimension.

**Figure 5:**
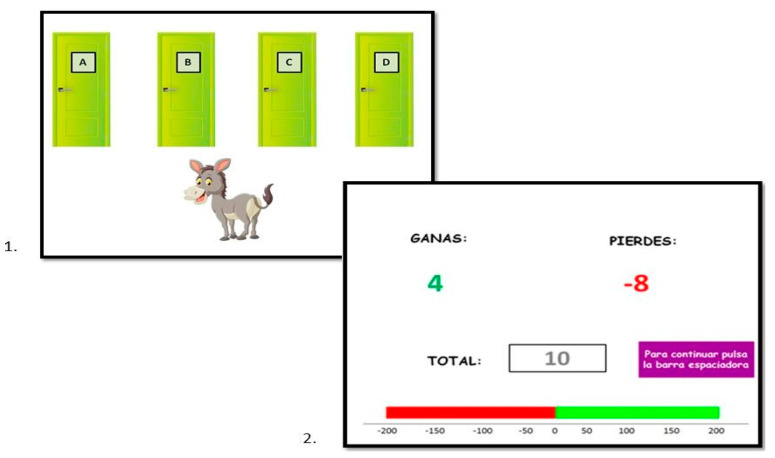
Example of a trial in which the participant chose door A, thus receiving a reward of 4 apples but a loss of 8 apples.

**Figure 6:**
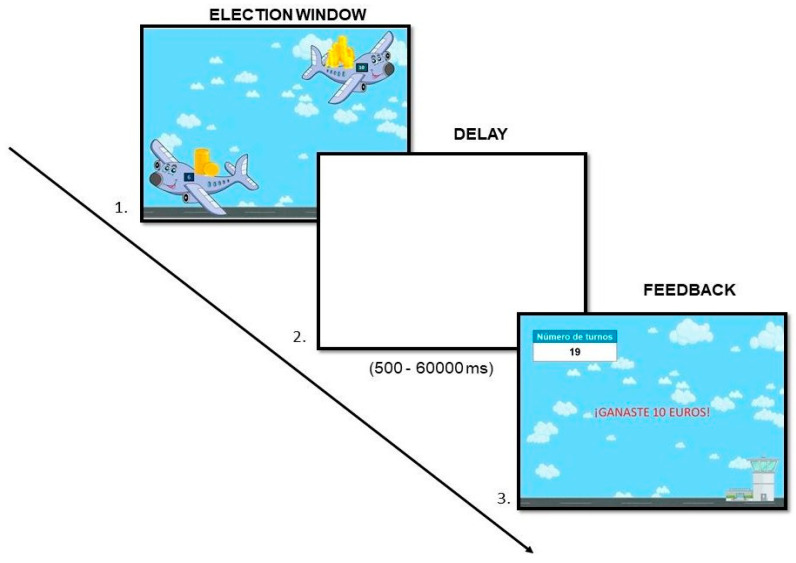
Example of a time delay discount trial. In this trial, participants have to choose between EUR 6 immediately or EUR 10 after a delay of 60,000 milliseconds.

**Figure 7:**
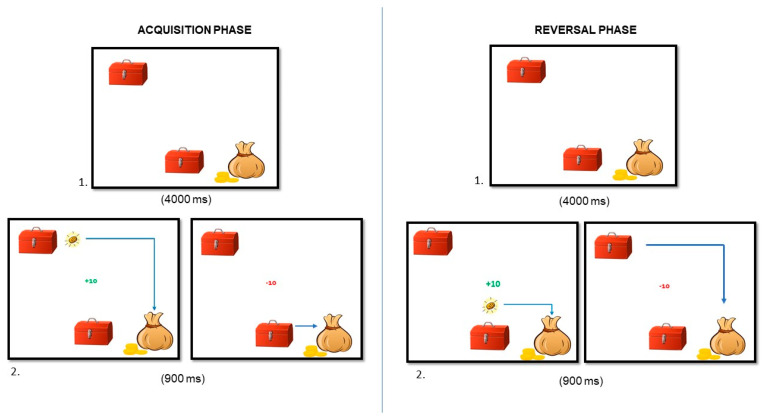
Acquisition and reversal phases of the affective reversal learning task.

- **The Colored Owls.** To evaluate *updating of working memory*, we designed an adapted version of the “2-back task” from Robinson and Fuller [42]. In this task, a sequence of colored owls is shown, and for each item participants are asked to catch the owls that present the same color as the one that appeared two trials before. Therefore, for each trial they must give a “yes” (Target trial) or “no” (No-Target trial) response, pressing “1” or “2” keys on the keyboard. This allows recording of the percentage of correct responses (yes response to a Target stimulus), as well as the percentage of false alarms (yes responses to a No-Target stimulus). Figure 1 shows the time exposure of stimuli in this task. In this task, participants first had to perform a practice block of 10 trials and two experimental blocks of 24 trials each (70% No-Target trials and 30% Target trials). From these data, the dependent variable calculated is the value of the parameter A’, applying the parameters of the Signal Detection Theory (SDT), which reflects the sensitivity of the participants to discriminate elements as they were presented (or not) in previous tests [43]. This index can range between 0 and 1, and when the value of this parameter is higher, it means that participants presented a good *updating of working memory* ability.

- **The Zoo Game.** This task evaluates the ability to inhibit an automated response adapted from the “Go/No-Go Task” from Lamm et al. [32,33]. At the beginning, participants are instructed to help the monkey called “Miguelito” “catch” (by pressing the space bar) all the animals that ran away from the zoo when they appear on the screen (GO trials), except when a picture of a monkey appears (No-GO trials) (see Figure 2 for a description of the time exposure of stimuli). The time interval between trials was variable (250, 500, 750, 1000, 1250, 1500, 1750, and 2000 ms). The task consists of a practice block of 12 trials, followed by 80 experimental trials (75% “Go trials” and 25% “No-Go trials”). For this study, the dependent variable is the percentage of errors of commission or false alarms (FAs) in the “No-GO trials”. Thus, a higher value of FAs will reflect a worse ability to *inhibit automated responses*.

- **Catch the Frog**. *Suppression of interference from distractors* was assessed using an adapted version of the “Flanker Task” from Traverso et al. [34,35]. In this task, participants are shown 5 frogs, presented in a horizontal line in the center of the screen. Participants are asked to help the frog in the center to jump. Therefore, they have to press the left or right mouse button depending on whether the frog in the center is facing left or right, regardless of whether the surrounding frogs are pointed in the same direction (congruent trials) or in the opposite direction (incongruent trials). In Figure 3, the time exposure from an entire sequence of stimuli is shown. This task consists of 4 practice trials and 48 randomized experimental trials (50% “congruent trials” and 50% “incongruent trials”). As the dependent variable, the interference effect (IE) is calculated from the difference in performance on incongruent versus congruent trials as a function of median response time (RT) and percent of errors. Thus, a high IE value indicates a worse ability to *suppress the interference from distractors*.

- **Find the Way.***Set-shifting* ability was evaluated using the Dimensional Change Card Sort (DCCS) task from the NIH Toolbox Cognition battery [36]. In this task, two stimuli are presented that vary in color (red or blue) and shape (drawing of a rabbit or a boat). Participants are asked to help the drawing presented in the middle of the trail (target) to choose the correct trail, selecting with the computer mouse one of the reference stimuli presented at the top of the screen based on the cue presented in each trial: either the word “color” or the word “shape”, which refers to the dimension according to which they had to classify the target. Figure 4 shows the time exposure for each event included in one trial. For two practice blocks, participants must classify according to one dimension or another (4 trials for each dimension). The experimenter supervises to make sure participants understand the instructions and are able to read properly the word to classify the target. Following this practice block, participants perform another two simple blocks without help. When children do not meet 3 of 4 correct responses, they have to repeat the block 3 times, and if they do not meet this criterion, the task ends. After passing the two simple blocks, a third mixed block of 30 trials is presented, where both dimensions are combined. Therefore, the participant must change their attentional focus between the two dimensions (80% “color” trials and 20% “shape” trials), where 6 trials are followed by a different classification dimension (“switching” trials). For this mixed block, participants do not have to meet any criterion to end the task. The dependent variables recorded in this task are the median response time (RT) and the percentage of errors in the simple and mixed blocks. From these measurements, the “Task-Switching Cost” (TSC) is calculated based on the RT and the percentage of errors. To do this, the value of the performance obtained in the task of “switching” trials from the mixed block and that obtained in the simple blocks of color and shape are subtracted. Thus, a high TSC on RT or percentage of errors is associated with a poorer *set-shifting* ability.

- **The Hungry Donkey.** *Decision making in situations of uncertainty* was assessed using a version of the “The Hungry Donkey Task” originally developed by Crone and van der Molen [37]. In this task, the child is presented in each trial with 4 identical doors on the computer screen (A, B, C, and D), and they must help the donkey choose the doors that provide the most points by pressing the computer keys C, V, B, and N, respectively (see Figure 5). The main characteristic of this task is that two of the doors presented (A and B) are considered disadvantageous because despite providing a higher profit after each choice (+4 points), after 10 trials the number of losses leads them to a negative total net score. Doors C and D are considered advantageous in providing a positive total score after 10 consecutive trials, although the gains after each choice are lower (+2 points). Another aspect in which the doors differ is in the frequency of losses associated with them. On the one hand, the choice of doors A and C leads to a higher frequency of losses (A: 8, 10, 10, 10, or 12 and C: 1, 2, 2, 2, or 3 losses every 10 trials). While, on the other hand, doors B and D imply a lower frequency of losses (B: a single loss out of 50 and D: a single loss out of 10). The dependent variable recorded for this task is the difference between the number of advantageous choices and the number of disadvantageous choices (NS: Net Score) in a total of 100 trials. Thus, a higher value of Net Score measure will reflect a higher *decision making under uncertainty* ability.

- **Planes and Rewards.** The *delay of gratification* ability was evaluated through a version of the delay discount task originally developed by Scheres et al. [44,45]. In this task, participants in each trial are shown two planes loaded with points and are asked to choose the plane they prefer, trying to earn the maximum number of points possible. In each trial they are always given the choice between an airplane with an immediate reward that can vary between trials (2, 4, 6, or 8 points) and another airplane that offers a greater reward (10 points) but that requires a variable timeout (5000, 10,000, 20,000, 30,000 or 60,000 ms). Participants must choose in 20 trials between the immediate reward by pressing the “I” key, or the delayed reward by pressing the “D” key on the keyboard (see Figure 6). The dependent variable recorded for this task is the area under the curve—AUC [46]. The AUC index describes the decrease in the subjective value of a reward when its availability is delayed. This AUC index can vary between 0 (steepest possible discount) and 1 (no discount). Therefore, a smaller AUC reflects a more pronounced discounting function, that is, a lower willingness to wait as the duration of the delay increases.

- **The Mechanic’s Game.** To assess *affective reversal learning*, we used an adapted version of the “Probabilistic Reversal Learning Task” by D’Cruz et al. [47], based on the probabilistic reversal learning (PRL) paradigm. Participants are told to help the mechanic to find which stimulus provides them coins, trying to gain as many coins as they can during the task. In each trial, when participants choose the stimulus considered the correct one, they gain “+10 points”. However, when they choose the incorrect stimulus, they lose “−10 points”. Participants are not told which of the two stimuli is the correct one but must discover it by exploring the options. See Figure 7 for the time exposure of each stimulus.

This task consists of two practice blocks and two experimental blocks (see Table 3 for a description of the main differences between these blocks). All four blocks consist of 10 consecutive trials, which can be repeated up to 6 times until participants meet the learning criterion described in Table 3. 

In practice blocks, choosing the correct stimulus is followed by the reward in 100% of the trials. The main difference between Block 1 and Block 2 of the practice blocks is that the correct stimulus changes to the other one when participants meet the learning criterion (9 consecutive correct answers). The stimuli presented in the practice blocks are two mechanic’s boxes. Once the learning criterion has been acquired, the participant must perform another two experimental blocks where the ratio of rewards and punishments changes to 80:20. That is, the stimulus considered correct in Block 1 of the experimental block is followed by a reward only in 80% of the trials, while in the remaining 20% the “incorrect” stimulus is rewarded. After fulfilling the learning criterion (8 consecutive correct answers), the correct stimulus changes, and the stimulus considered in the previous block as correct becomes incorrect and provides rewards only in 20% of the trials. 

For this task, three types of dependent variables that occur in the second experimental block were recorded: (1) perseverance errors that occur when, although the inversion of contingencies occurs, and the correct answer becomes the other image, the participant continues to choose the answer that was correct before; (2) regression errors that occur when, after having chosen the new correct image, participants again choose the now incorrect image; and (3) switching errors, which occur when negative feedback is provided despite having selected the correct image; on the next try, the participant chooses the other incorrect image.

**Figure 8:**
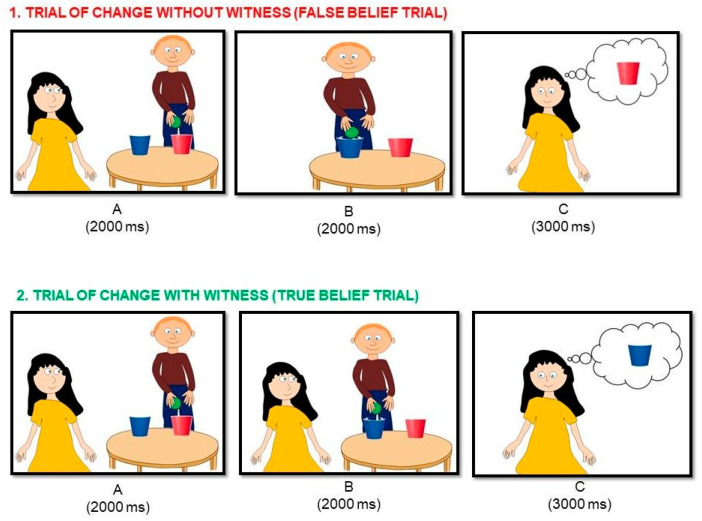

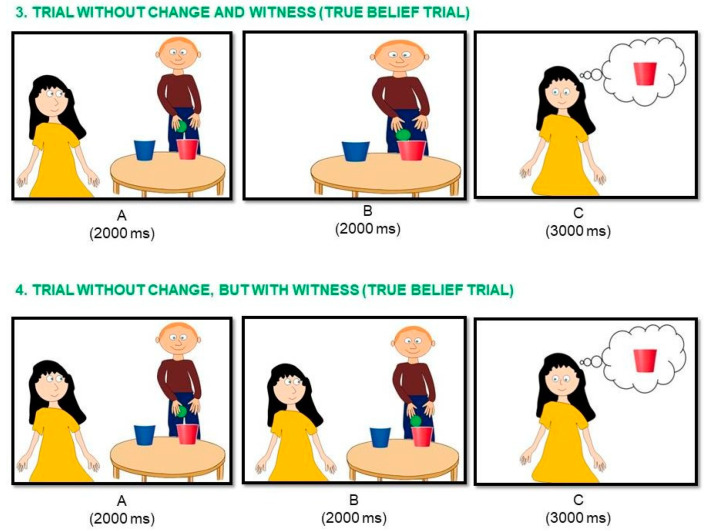
An example of the images of Jack and Maria for the four types of trials. In all these examples, the correct answer is “yes”.

- **Playing with Jack.** To assess *theory of mind*, and more specifically the ability to understand false beliefs, we used the “Jack and Jill Task” by Dennis et al. [48]. In this task, in each trial the character named Jack hides the ball in a blue or red glass, and the participant must indicate where the character of Maria will think the ball is hidden. In total, we can find 4 types of trials depending on whether Jack changes the ball of the glass (“change or no change trials”) and if Maria is present when he changes the ball (“witness or non-witness trials”). Figure 8 shows the time exposure to each stimulus shown in the task as well as the four conditions mentioned above. The task consists of a practice block where participants are presented 4 trials of each condition, and the experimenter ensures that they have understand the instructions. Then, there is an experimental block in which they have to respond as quickly as possible as to where Maria will look for the ball. The “non-witness and change” trials are considered a measure of understanding false beliefs (called “ToM trials”), as they require the participant to understand the belief that Maria has, that is, the location of the ball will be wrong because they will not have seen how the ball moved. Therefore, the percentage of accuracy in this type of “ToM trials” is recorded for this study, with a higher score showing that children have a greater ability to understand false beliefs.

#### 2.1.3. Procedure 

Before the study was carried out, approval was obtained from the Human Research Bioethics Committee of the University of Almería (Spain), respecting the ethical principles of the 2013 Helsinki declaration and other international codes. In addition, before their participation, the parents of the children gave written informed consent.

The different tasks were administered to the participants individually in a separate and quiet room of the school during the morning hours. The mean time to complete all the EBB tasks was approximately 60 min, with the necessary breaks in case the participant began to show signs of fatigue. 

Both the order in which the EBB tasks were administered (using a laptop) and the instructions were the same for all participants. In the first part of the evaluation, all the cool EFs tasks were presented and, before starting the block of hot EFs tasks, the participants were informed that in the next four tasks they had to try to gain as many points as possible in order to participate in a raffle for a surprise prize when the project was finished. This was done with the aim of ensuring that the tasks designed to evaluate the hot components of EFs set in motion those “hottest” mechanisms, thus achieving the more emotional and motivating context in which these components are usually activated. For this reason, it was not possible to counterbalance the order of presentation of the cool and hot tasks, since presenting the contest first and the hot tasks associated with the loss or gain of points could have activated some ventromedial regions from the prefrontal cortex (region associated with processing reward gains and losses), which could have interfered with the abstract and more “cool” character of cognitive tasks.

#### 2.1.4. Statistical Analysis

Preliminary analyses included gender and parental educational level as variables to explore their potential impact on the performance of our sample in cool and hot EF tasks. Since none of these variables were significant, gender and parental educational level were not included in subsequent analyses.

We first examined the normal distribution of variables using the Shapiro–Wilk test to assess age-related changes in cool and hot EF measures. Since all variables exhibited a normal distribution, we performed a one-way ANOVA for each hot and cool EF measure, with the age group as the between-subject factor (6, 7, 8, 9, 10, and 11 years old). Post hoc tests were carried out using the Bonferroni method for multiple comparisons in cases where significant effects were identified. Statistical significance was set at *p* < 0.05. All statistical analyses were conducted using SPSS Version 26 (IBM Corp., Armonk, NY, USA).

### 2.2. Results

#### 2.2.1. Feasibility of the Tasks Included in the Executive Brain Battery (EBB)

As seen in Table 4, in each age group, at least 83% of the participants could complete all the tasks included in the EBB, while 89% of the total sample was able to complete all EBB tasks.

For the **cool EF tasks**, three out of four were deemed unfeasible for some children in the 6, 7, 8, 9, and 10-year age groups. “The Zoo Game” (*inhibition of an automated response*) and “Catch the Frog” (*suppression of interference from distractors*) could not be completed by five children aged 7, 9, and 10, as they did not pay attention to the experimenter’s instructions, stopped mid-task, or encountered difficulties with the use of the computer mouse. Regarding “Find the Way” (*set shifting*), 21 children aged 6, 7, 8, 9, and 10 could not achieve the minimum number of correct responses required to complete the task. Specifically, these children faced challenges in providing four correct responses out of the five trials in each of the simple blocks, where they had to classify based on the color or shape of the target. Participants had the opportunity to repeat the same block up to three times to meet the criterion. However, as they did not achieve the minimum correct responses required, these 21 children were considered outliers [36].

Regarding the four **hot EF tasks**, “The Mechanic’s Game” (*affective reversal learning*) could not be completed by 17 children aged 6, 7, 8, 9, and 11 years due to not paying attention to the experimenter’s instructions (*n* = 2) or not reaching the minimum number of correct responses required to complete the task (*n* = 15). 

#### 2.2.2. Age-Related Changes in the Cool and Hot EF Tasks 

The means and standard deviations obtained for each age group in each hot and cool task and the results of the one-way ANOVA are displayed in Table 5 and Table 6.

Regarding the cool EF tasks, as depicted in Table 5, age significantly influenced the measurements obtained in *updating of working memory*, *interference suppression*, and *set shifting*, with improved performance observed as the age increased. In the *C* task (“The Colored Owls”), post hoc analyses revealed that the younger children’s group exhibited significantly lower scores compared to the 9-year-old group (*p* < 0.001). Similarly, in the interference suppression task (“Catch the Frog”), concerning the interference effect with the percentage of errors, statistically significant differences were noted between the youngest children’s group and the 7-year-old group (*p* < 0.05), with older children demonstrating better performance. In the set-shifting task (“Find the Way”), it was also observed that for the task-switching cost (measured in the percentage of errors), the 8-year-old group displayed a higher task-switching cost compared to the 6-year-old group (*p* < 0.05).

Concerning the hot EF tasks (see Table 6), age exhibited a significant effect solely on the performance of the *theory of mind* task (“Playing with Jack”). A higher percentage of correct answers was observed as the age group increased. Post hoc analyses for this task revealed statistically significant differences in the percentage of correct answers between the younger children’s group and the 11-year-old group (*p* < 0.05).

### 2.3. Discussion 

The results from this first study highlight the viability of the EBB in assessing both the cool and hot components of EFs during middle childhood. As anticipated, the tasks appear suitable for children aged 6 to 11, with 83% of the study participants completing them. The primary cool EFs task that posed difficulty for children was the “Find the Way” task, where 21 out of 195 participants encountered difficulty classifying stimuli based on their color or shape in simple blocks. These results may be notable, as the “Find the Way” task was designed based on the well-established “DCCS” from the “NIH Toolbox Cognition” battery [36], which is a more demanding task derived from Zelazo’s original “Dimensional Change Card Sort” [49]. The key distinction lies in the fact that in the standard version, children only need to classify stimuli according to one dimension and then another dimension without a minimum correct response requirement. In contrast, the “DCCS” from Zelazo et al. [36] necessitates at least 4 out of 5 correct responses to complete the task. Initial studies with this more challenging task from the “NIH Toolbox” battery have suggested that this new version could be more sensitive to age-related changes in middle childhood. However, limited research has explored age-related changes in middle childhood using this version of the “DCCS”. Consequently, future research analyzing this task with typical development will be valuable in clarifying its sensitivity to age-related changes. 

On the other hand, the hot EF task that proved more demanding was “The Mechanic’s Game”, assessing *affective reversal learning* ability. This task was designed based on the “Probabilistic Reversal Learning” task developed by D’Cruz et al. [47]. Although this type of task has been more commonly utilized with clinical populations (e.g., [47,50], there is a scarcity of studies involving children with typical development in middle childhood [40]. Moreover, variations in the design of each task make direct comparisons regarding age-related changes challenging. For instance, Van Duijvenvoorde et al. [40] designed a task where children choose between four boxes associated with various pictures (e.g., banana—A; sun—B; bike—C; flower—D). In each block, two of these boxes are presented, and choosing one (e.g., A) is associated with a gain in 70% of 10 trials and a loss in 30%. Another box (B) provides a gain and loss in the opposite ratio (30:70) for ten trials. This first block is presented only once, regardless of the number of correct answers the children provide. Subsequently, children are presented with two different boxes (C and D) with the same gain and loss ratios as the A and B boxes but presented in different positions on the screen, assessing their ability to recognize that the stimulus located in the previous position is no longer the “correct” one. Children are not required to meet any correct response criterion for this second block.

As evident, our task, “The Mechanic’s Game”, differs substantially from the task used by Van Duijvenvoorde et al. In our task, the same stimuli were consistently presented in the experimental block (two identical toolboxes). Participants were required to provide a minimum of eight correct answers for learning to be considered as having occurred (regarding which box was deemed “good” on account of yielding the most points). Therefore, while both tasks are considered to assess *affective reversal learning* ability due to an inversion of the learned contingencies associated with two stimuli, differences in other aspects of the tasks could explain why we found varying results in age-related changes in this ability as well as the observed difficulties in performing this task in 9% of the sample.

The findings also suggest that in middle childhood, there are more age-related changes in cool EFs than in hot EFs, aligning with observations made by other authors [51,52]. Specifically, all cool EFs can show significant improvements between ages 6 and 9, except for the ability to *inhibit automated responses*, where no significant change was observed between ages 6 and 11 using the task in our EBB. Among the hot EF tasks, the only one indicating age-related changes within this age range is the ability to understand false beliefs, which shows improvement from ages 6 to 11. However, the other hot components of EFs showed no significant age-related changes. Despite this, the fact that all age groups could perform these hot tasks suggests that tasks included in the EBB are suitable for assessing these hot components.

## 3. Study 2

The primary objective of this study was to investigate whether, at the end of middle childhood (ages 11–12), children could demonstrate performance similar to that of adults in both the cool and hot EF tasks included in our *Executive Brain Battery* (EBB). Consequently, in this study, we compared the performance on the EBB of the 11-year-old children who participated in the previous study with that of a group of adults. Considering the limited existing studies that directly compare children’s and adults’ performance in cool and hot EF tasks, in conjunction with the results of our first study, we anticipated observing an adult-like performance by the age of 11, particularly in the cool tasks.

### 3.1. Materials and Methods

#### 3.1.1. Participants 

Thirty adult volunteers aged between 25 and 35 (M_age_ = 28.33, SD = 3.04, 53.33% female) were recruited from social media advertisements. Moreover, none of the participants had any neurological or psychiatric disorders.

#### 3.1.2. Measures 

For this second study, we employed the same eight EEB tasks utilized in the first study. Additionally, to ensure that the hot EF tasks were as engaging and emotionally resonant for the adults as they were for the children, participants were informed before starting this block of tasks that there would be a raffle for a surprise prize, based on the total points achieved during the session.

#### 3.1.3. Procedure

The assessment was conducted in the Basic Psychology Laboratory. The volunteers gave their written informed consent to participate, and all participants received the same instructions as the children. 

#### 3.1.4. Statistical Analysis

For the “Find the way” task, which assesses *set-shifting* ability, one adult was excluded from the analysis because he was a clear outlier for his age (i.e., failing to meet the accuracy criterion for simple blocks), resulting in a final sample of n = 29 for this measure.

To determine whether children at the age of 11 show performance comparable to adults on each cool and hot EF measure, we conducted parametric Student’s t-tests, comparing scores between the 11-year-old and adult groups for each measure.

### 3.2. Results

Table 7 and Table 8 show the results obtained in the EBB by the 11-year-old and adult groups.

Regarding **cool EF tasks**, a statistically significant difference was only observed between the groups in the *updating of working memory* task (*p* < 0.001), with adults demonstrating better performance than children. However, no statistically significant differences were found between children and adults for the remaining cool EF tasks included in our EBB (*inhibition of automated responses*, *interference suppression*, and *set shifting*) (see Table 7).

For **hot EF tasks**, as can be seen in Table 8, in the *affective reversal learning* task, children made fewer regression errors than adults, with this difference being marginally significant (*p* = 0.05). In contrast, in the *theory of mind* task, the adult group performed significantly better than the children (*p* < 0.05). Moreover, the results for the *decision-making* task indicated that the adults also made better decisions than children (although the differences were marginally significant; *p* = 0.06). Additionally, no significant differences were found between adults and children on the *delay of gratification* task. 

### 3.3. Discussion 

The results obtained in the present study suggest that the core cool EFs, such as *inhibition of an automated response*, *set shifting, and interference suppression*, could reach an adult-like level at the end of middle childhood. However, other core cool EFs, such as *updating of working memory*, may continue to differ from adults until the age of 11, as observed in the study by Schleepen and Jonkman [53]. Regarding the hot EFs explored in this study, the findings imply that at the end of middle childhood (around 11–12 years), only *theory of mind* (specifically, the ability to understand false beliefs) does not appear to have reached a level similar to that of adults. 

It is noteworthy that, while our study did not reveal statistically significant differences between children and adults in various cool and hot EF tasks (*inhibition of automated responses*, *interference suppression*, *set shifting*, *affective reversal learning*, *decision making under uncertainty*, and *delay of gratification*), we cannot conclude that these EFs are fully developed by the age of 11–12. Age-related changes can extend throughout the entire lifespan, as some authors have noted that these cool EF abilities continue to evolve from age 35, observing a decline until the end of adulthood [54]. Consequently, it would be insightful for future studies to include a broader age range, exploring changes in EFs using an approach similar to that of Ferguson et al. and incorporating tasks to investigate changes in hot EFs beyond middle childhood.

## 4. General Discussion

The current work comprises two studies examining the feasibility of the *Executive Brain Battery* (EBB) to assess age-related changes in cool and hot EFs during middle childhood. Additionally, it aims to explore whether children would exhibit adult-like performance on each cool and hot EF task from the EBB at the end of middle childhood.

One of the main findings to emerge from the first study is that the EBB appears suitable for use with children aged 6 to 11, with approximately 89% of participants completing all tasks included in the battery. Some limitations were observed in the “Find the Way” task designed to evaluate *set-shifting* ability. The “Find the Way” task in our EBB is based on a modified version of the “Dimensional Change Card Sort” (DCCS) developed by Zelazo et al. [36]. This modified version differs from the original DCCS by Zelazo [49] in that, in the modified version, children must achieve a specific number of correct answers (4 out of 5) to proceed with the task. Failure to do so renders the task impracticable. In contrast, in the original version, children do not need to meet a minimum correct response criterion to pass the simpler task.

The results of the present study indicate that the “Find the Way” task was impracticable for 11% of the sample due to difficulties in classifying stimuli solely based on their color or shape. Although setting a criterion of four correct answers out of five trials for each simple block may seem demanding, Zelazo et al. [36] emphasized that this criterion is not overly challenging, as Zelazo [49] observed that children can successfully classify a stimulus according to color or shape from the age of 5. In their feasibility study of the modified version of the DCCS, Zelazo et al. [36] found that between ages 3 and 15, 20% of their sample could not meet this criterion (four of five correct responses when classifying by color or shape). In the present study, only 11% of the sample could not achieve this criterion. Additionally, another study with adults [55] demonstrated that this modified version of the “DCCS” is sensitive to age-related changes across adult ages. In particular, they found that set-shifting ability could improve until age 29, after which it declines until age 86. These findings suggest that the observed results in our study may not be concerning, and this modified version of the “DCCS” included in the EBB could be useful for comparing *set-shifting* ability changes across the lifespan.

Nevertheless, considering the difficulties observed in our sample of children with “typical” development, some adaptations that could be made to the “Find the Way” task to enable its use in populations with delays in language development include (1) using pictograms to indicate the classification dimension, instead of the words “COLOR” or “SHAPE” used in the task or (2) increasing the number of trials in the simple blocks and thus increasing the likelihood of participants fulfilling the correct response criterion by incorporating more trials. This approach would allow participants more training to become familiar with the demands of the task. 

Another challenging task for children between 6 and 11 is “The mechanic’s game”, assessing *affective reversal learning* ability. The results indicated that 7% of the sample had difficulties meeting the criterion of eight correct responses on each block of 10 trials, even with six attempts allowed. However, it is essential to consider that this hot EF has been minimally studied in middle childhood with “typical” development [12]. To our knowledge, only one study has explored age-related changes between 8 and 24 years in this hot EF using the “Probabilistic Reversal Learning” task [40]. However, in this task, no minimum number of correct responses was established within the experimental blocks to pass the task. Therefore, this might be one of the possible reasons why Van Duijvenvoorde et al. did not find difficulties in performing this task in their sample. Nonetheless, we do not consider this a limitation of the task since, without a minimum correct response criterion, we cannot ensure that participants genuinely learned the correct response, even if it sometimes resulted in losses. Thus, we cannot verify whether participants unlearned the initial stimulus–response association, learned the reversed contingencies, and adapted their choices for a more advantageous outcome. Moreover, as observed with the “Find the Way” task, it seems that “The Mechanic’s Game” task could be useful for assessing age-related changes across the lifespan [40,56]. These findings are supported by the differences observed at the end of middle childhood (11 years) compared to a group of adults, suggesting that this task is suitable for comparing various age groups. 

Regarding the sensitivity of the EBB to detect age-related changes in middle childhood, more substantial changes were observed in the cool EF tasks than in the hot EF tasks between 6 and 11 years, consistent with findings of Hooper et al. [51] and Prencipe et al. [52]. However, the absence of significant age-related changes in some tasks does not imply that these tasks are unsuitable for evaluating EFs in this age range. On the contrary, these results might indicate no substantial improvements in EF abilities during this stage of development, particularly in most hot EFs, possibly due to the later maturation of certain prefrontal cortex (PFC) regions. For instance, Steinbeis et al. [57] suggested that the lack of changes between 6 and 12 years in the ability to *delay gratification* (measured similarly to our EBB task) could be attributed to the late maturation of the ventromedial prefrontal cortex (vmPFC). In their study, children were able to recognize that a larger reward was more valuable, yet they consistently chose smaller but immediate rewards.

Likewise, in this study, we also found that in most of the cool and hot EF tasks, at the end of middle childhood, most children showed a level of performance similar to that of adults, except for *updating of working memory* and *ToM* tasks. These results suggest that the tasks included in the EBB to assess these three EFs might be useful for comparing age-related changes in a broader age range than between 6 and 11 years. However, this notion should be investigated further in future studies with a more extensive sample. 

Below, we will discuss the age-related changes found for each cool and hot EF explored with our EBB.

### 4.1. Cool Executive Functions

**Updating of working memory**. Improvements in this ability were observed between the group of younger children and 9-year-old children. However, the 11-year-old group did not exhibit similar adult-like performance in this cool EF task. Our pattern of results aligns with observations from previous studies [31,53,58].

**Inhibition of an automated response**. The results observed in our study regarding the ability to inhibit automatized responses suggest no substantial changes during middle childhood, as we did not find an age-related effect in the task assessing this cool EF. Additionally, there were no performance differences in this cool EF task between 11-year-old children and the group of adults. However, some previous studies have reported significant improvements between 6 and 11 years old [51]. Symeonidou et al. [59] found that at age 11, children continued to show lower performance compared to adults. These discrepancies between the results of our study and previous findings could be explained, in part, by differences in the tasks used. For instance, the task employed by Hooper et al. [51] may be influenced by the time of exposure to “Go” and “No-Go” stimuli, with participants having only 250 ms to respond. In contrast, participants had more time (1500 ms) in our task. Additionally, the version used by Symeonidou et al. [59] required participants to indicate the side on which a square appeared on the screen only when it was a green square (“Go” trials), not a red one (“No-Go” trials). The demands of this task included inhibiting responses and a higher working memory demand, suggesting that modifying some task parameters could enhance its sensitivity to age-related changes. In this regard, other studies employing a simpler version of the “Go/No-Go” task did not find significant age-related changes in middle childhood [60,61]. Additionally, concerning the comparison between children and adults, these studies also observed that 11-year-old children did not exhibit an adult-like performance level in simpler “Go/No-Go” tasks [61,62].

**Suppression of interference from distractors**. The ability to suppress distractor interference shows significant improvements in this development period. Specifically, there is a noticeable decrease in errors made when resolving conflicts introduced by distractors between the ages of 6 and 7. Moreover, the interference effect appears to diminish significantly after age 7, as no differences are observed up to 11 years of age nor between age 11 and adulthood. Similar results have been found in other studies utilizing tasks akin to the ones employed in the present study [63,64,65,66].

**Set shifting**. Concerning set-shifting ability, our results indicate an increase in the percentage of errors due to switching attention between different target dimensions, particularly between the ages of 6 and 8. Although it seems that by the end of this developmental stage, children exhibit similar performance to adults on the task used in this study, this contrasts with findings from prior studies employing the “DCCS” task [65,67,68]. The variability observed in the scores of the 8-year-old age group regarding the percentage of errors might contribute to these disparate results. However, given that this poorer performance is solely evident in the percentage of errors (and not response time), caution is warranted when interpreting these findings. Future studies with a larger participant pool in each age group would help to clarify these results. Moreover, Ezekiel et al. [69] also noted that by age 11, children showed an adult-like performance on the “DCCS”. The absence of differences found between 11-year-old children and adults in the performance of this task does not imply that it is unsuitable for detecting age-related changes after 11 years. As previously mentioned, EFs undergo constant changes throughout life. Thus, future studies incorporating a broader age range encompassing adolescence and early adulthood would help determine the sensitivity of this task to detect changes across the lifespan. Furthermore, none of these studies, which yielded results similar to the present study, imposed minimal correct responses to pass the task, as applied in the “Find the way” task. Consequently, these researchers did not encounter issues of impracticability in their samples. As Chevalier and Blaye [68] suggested, future studies could benefit from additional practice blocks, including a mixed block, to ensure children comprehend the task. Additionally, these authors utilized pictograms displayed throughout the trial instead of the words “COLOR” or “FORM” used in the original version of the “DCCS” [49]. These adaptations do not impact the task’s sensitivity, achieving more practicability in the sample.

### 4.2. Hot Executive Functions

**Decision making under uncertainty**. This study found no significant differences between the 6 and 11 age groups in decision making under uncertainty. Our results indicate that by age 11, children exhibit an adult-like level of performance on “The Hungry Donkey” task. However, the results for this task could vary depending on the study design. For instance, other cross-sectional studies similar to ours did not find significant differences between the 6 and 11 age groups [37,38]. In contrast, Lensing and Elsner [13] reported age-related changes (between 7 and 11 years old) in their longitudinal study. They emphasized that longitudinal studies enable exploration not only of linear changes in the development of cognitive processes but also of curvilinear, quadratic, and linear development trajectories. Due to the limited number of studies examining this aspect of EF in middle childhood (to our knowledge, only Lensing and Elsner have addressed this issue), it is crucial to continue conducting studies with both cross-sectional and longitudinal designs to further explore age-related changes in this hot EF. Contrary to our results, these other studies [37,38] have suggested that at the end of middle childhood, children might not yet have reached a level comparable to adults in the *decision-making* task. Nevertheless, our study found marginally significant differences between the two age groups, with adults performing higher on this task. While these differences did not reach significance, they could imply that the performance in the two groups was quite similar. Increasing the number of participants in each group could reveal significant differences. 

**Delay of gratification**. The results regarding the ability to delay gratification also did not reveal any significant changes between ages 6 and 11, and children exhibited an adult-like level in the delay of gratification task by the age of 11. In contrast, previous studies [52,57] have highlighted significant age-related differences in how children devalue large rewards that require waiting. Limited studies have compared age-related changes in delay of gratification ability using a task similar to our “Planes and Rewards” task in middle childhood. Some of these studies [70,71] did not find significant changes in this hot EF ability between 8 and 11 years old. The disparate results may be linked to the motivational factor, as studies where children showed significant changes with age often involved being paid the reward they earned at the end of the task [52,57]. Therefore, the motivational element could influence task performance and might explain why we did not find significant differences between the 11-year-old group and adults, which is consistent with Burns et al. [71]. 

**Affective reversal learning**. Despite being a fundamental executive function, *affective reversal learning* ability did not show any significant change in the 6- to 11-year-old group. However, by the end of middle childhood, children showed marginal differences in the percentage of regression errors in “The Mechanic’s Game” task compared with the adult group. This result suggests that when the proportion of gains/losses changes in the second experimental block, adults tend to select more frequently the stimulus that previously provided more gains, even when selecting it now results in more frequent losses. Weiss et al. [56] explained that to perform tasks involving *affective reversal learning*, the ability to use new information to update internal beliefs about stimulus–reward associations is crucial, leading to efficient learning in unstable environments. The authors suggested that this updating mechanism may contribute to a reduction in error rates, particularly regression errors, with age. However, updating information “too quickly” by changing response options after negative feedback could also lead to more errors, with fewer conservative answers. Weiss et al.’s [56] study explored this ability in children aged 8 to 12 and adolescents aged 13 to 17, revealing a decrease in regression errors with age. However, this study did not investigate how this ability continues to change beyond late adolescence, emphasizing the need for future research to provide a more comprehensive understanding of its development during middle childhood. 

**Theory of mind**. In contrast to other hot EF components, *Theory of Mind* (ToM) is the only hot EF showing significant developmental improvement during middle childhood. The current findings indicate a substantial enhancement in understanding first-order false beliefs, as measured by the “Playing with Jack” task, between 6 and 11 years of age. Notably, there are significant differences between children and adults at the end of this period. These results build upon earlier observations that this ability improves markedly between 6 and 9 years of age [14,72]. While false belief tasks are often considered the primary assessment tools for *ToM* in children [73], *ToM* is a broad and diverse construct encompassing various abilities with different developmental trajectories [74]. It is crucial to emphasize that the results presented in this study specifically focus on the automatization of the ability to understand false beliefs, as children typically demonstrate correct responses to another person’s false beliefs by the age of 5.

Regarding age-related changes during middle childhood in a population with “typical” development, the results of our study show how during this developmental period there seem to be more age-related changes in cool EFs than in hot EFs. Specifically, age-related changes appear to occur between ages 6 and 7 in the ability to suppress interference from distractors, between ages 6 and 8 in the ability to set shift, and between ages 6 and 9 in the updating of working memory. In contrast, for the inhibition of prepotent responses, we did not find any improvement during middle childhood. Taking into account the age-related changes observed in the present study and the results found in previous studies, we hypothesize that within cool EFs, the results indicate that the ability to inhibit automated responses matures mainly during early childhood, showing no significant changes between 6 and 11 years of age. This ability to inhibit automatized responses could underpin changes that occur at age 7 in the ability to suppress interference, which requires inhibiting the natural tendency to respond to more salient stimuli and instead focus attention on the main task or objective. In parallel, set-shifting ability continues to improve until age 8, while the two types of inhibitory control seem to show no change until age 11. Therefore, this ability may depend on a certain level of improvement in the ability to suppress interference that may be caused by the irrelevant dimension after the change of instructions (or task) as well as to inhibit the response to the dimension that was previously relevant and had become automated. At the same time, from 6 to 9 years of age, the updating of working memory ability appears to be changing, with a prerequisite for its optimal functioning being the ability to inhibit content that is no longer relevant according to the demands of the task as well as to flexibly replace obsolete information based on new relevant information. 

In contrast, in hot EFs, the only significant change that was observed between 6 and 11 years was in theory of mind, which improved throughout middle childhood. Based on the current results, affective reversal learning and delay of gratification appear to be more basic skills for which significant manifestations and changes have been observed in developmental stages prior to middle childhood. For example, children between 3 and 4 years old are able to resist the temptation to eat candy to receive a larger amount after a certain waiting time, as demonstrated by the marshmallow task, commonly used in early childhood to evaluate delayed gratification [75]. In contrast, first-order false belief understanding (one of the key theory of mind abilities) appears to show significant changes across middle childhood. This could be, as some authors have already pointed out, because the emergence of theory of mind seems to depend on the sufficient maturation of certain EFs [76]. More specifically, Austin et al. [76] observed that understanding first-order false beliefs requires the ability to suppress one’s own knowledge about reality and, thus, change one’s perspective to be able to put oneself in another’s shoes. During this change, we must maintain and update in our mind the relevant information about the reality we know and the reality that the other person knows, in order to detect the other’s false belief. Finally, it appears that the most important changes in decision making under conditions of uncertainty may occur during adolescence, especially between ages 15 and 16 [37]. Therefore, decision making in a situation of uncertainty could be an example of a more complex executive component that requires the prior development of other EFs to reach an optimal level. These predictions must be contrasted in exploratory or confirmatory factor analyses, where measures of all these executive components are included and which cover a broader age period that allows us to know whether some of these EFs are already developed at the beginning of childhood or whether, on the contrary, the most important changes occur in adolescence.

## 5. Limitations and Final Conclusions

Despite the valuable insights gained regarding the sensitivity of our EBB to assess age-related changes in cool and hot EFs between ages 6 and 11, it is important to interpret our findings considering several limitations. First, we could not assess some relevant socio-demographic variables (such as culture, ethnicity, native language, or socioeconomic status, to name a few). Neither could we access different schools from different cities to explore the viability of the EBB. Therefore, it is necessary to carry out more studies that let us explore how these socio-demographic differences could affect the viability of the EBB as well as age-related changes in EFs in children between 6 and 12.

Second, the absence of an adolescent group limits our understanding of the developmental changes that occur from middle childhood to adulthood. This period involves significant reorganization of prefrontal systems, marked by the peak volume of gray matter in the prefrontal cortex during adolescence [77]. Additionally, the neuronal reorganization is influenced by social and academic experiences during this stage [10]. Consequently, it would be useful to conduct studies that address this crucial developmental phase, considering both cognitive and socio-emotional aspects of executive functions. 

Only a few studies have directly compared children and adult groups in executive function tasks. One possible explanation for this scarcity might be the challenge of comparing cognitive abilities across different tasks with varying complexities and formats. An alternative approach could involve designing adaptive or progressive tasks, wherein the difficulty level is adjusted based on individual performance. This could mitigate potential ceiling and floor effects as encountered in traditional task designs. However, our study demonstrated that tasks included in the EBB did not show ceiling or floor effects, as even adults did not reach maximum scores in most tasks.

However, it could be interesting to use adaptive tasks that would also allow us to carry out longitudinal studies where we can evaluate children at different time points in order to describe the development trajectories followed by cool and hot EFs; since cross-sectional designs allow for more limited analyses.

Despite these limitations, the present study developed a comprehensive battery of executive tasks based on a holistic model of EFs, emphasizing the importance of assessing EFs in both cognitive abstract contexts and those with high emotional, motivational, or social loads.

For this reason, the present work aimed to provide an in-depth analysis of whether these tasks are sensitive to age-related changes in middle childhood while also considering the limitations inherent in evaluating children aged 6 to 11 years and the potential adaptations for the different child populations being studied. Furthermore, this study seeks to offer data that can serve as a reference for studies directly comparing children with typical development and those with developmental disorders. 

Consequently, we anticipate that the results of this study will have significant implications for educators, clinicians, and researchers working in the field of executive functions in both typical and atypical populations. The tasks included in the EBB were designed with minimal verbal content so that they could also be used in child populations with alterations in language development (such as most cases of deafness, Specific Language Disorder, or Dyslexia). This battery can be especially useful for those professionals who need tools sensitive to these linguistic limitations when studying the executive profile of children. Therefore, this battery offers the advantage of being able to control, to a certain extent, linguistic bias, which has been seen in the scientific literature to affect the executive functioning of oral deaf children [78] as well as children with Autism Spectrum Disorder [79], Specific Language Disorder [80] or Attention Deficit Hyperactivity Disorder [81].

In this sense, in future studies, this tool could help us describe the executive profiles of different childhood populations, thus allowing for the design of interventions and curricular adaptations based on the specific needs of each child. To do this, it is necessary to continue exploring this battery in different child populations to allow us to make the appropriate improvements and adaptations to achieve the objective of developing a sensitive battery to compare child populations with different linguistic alterations.

We have made access to our *Executive Brain Battery* freely available through the following website: (http://www2.ual.es/multisensory-brain/es; Accessed on 25 July 2024).

## Figures and Tables

**Table 1:** Sociodemographic data for each age group.

Age Group (Years)	6 (*n* = 20)	7 (*n* = 30)	8 (*n* = 37)	9 (*n* = 48)	10 (*n* = 34)	11 (*n* = 26)	All (*n* = 195)
**Gender** *f* (%)							
Male	12 (60)	13 (43.3)	19 (51.4)	28 (58.3)	17 (50)	7 (26.9)	96 (49.2)
Female	8 (40)	17 (56.7)	18 (48.7)	20 (41.7)	17 (50)	19 (73.1)	99 (50.8)
**Parental Education** *f* (%)							
Basic	1 (5)	6 (20)	7 (18.9)	11 (22.9)	15 (44.1)	8 (30.8)	48 (24.6)
Medium	7 (35)	7 (23.3)	15 (40.5)	20 (41.7)	6 (17.7)	7 (26.9)	62 (31.8)
High	11 (55)	16 (53.3)	12 (32.4)	17 (35.4)	12 (35.3)	10 (38.5)	78 (40.0)
Unknown	1 (5)	1 (3.3)	3 (8.1)	-	1 (2.9)	1 (3.9)	7 (3.6)

**Table 3:** Description of the main characteristics of “The Mechanic’s Game” task.

Tasks Blocks	Learning Criterion	Ratio of Rewards/Punishments of CR	Type of Stimuli
Practice blocks	9/10 consecutive CRs	100/0	Mechanic’s boxes
*Block 1*			
*Block 2*			
Experimental blocks	8/10 consecutive CRs	80/20	Blue cars
*Block 1*			
*Block 2*			

*Note.* Correct Responses—CRs.

**Table 4:** Percentage of participants who completed each task of the EBB according to age group.

Executive Functions	6 (*n* = 20)	7 (*n* = 30)	8 (*n* = 37)	9 (*n* = 48)	10 (*n* = 34)	11 (*n* = 26)	Total (*n* = 195)
**Cool Tasks**							
*The Zoo Game*	100	96	100	96	94	100	97
*Catch the frog*	100	93	100	96	97	100	97
*Find the way*	85	83	92	83	94	100	89
*The colored owls*	100	100	100	100	100	100	100
**Hot Tasks**							
*Hungry Donkey*	100	100	100	100	100	100	100
*Planes and Rewards*	100	100	100	100	100	100	100
*The mechanic’s game*	90	90	86	88	100	96	91
*Playing with Jack*	100	100	100	100	100	100	100

**Table 5:** Means and standard deviations for each age group for cool EF tasks.

	Age Group							
Cool Executive Function	6 *n* = 20	7 *n* = 30	8 *n* = 37	9 *n* = 48	10 *n* = 34	11 *n* = 26	*F*	*p*
***Updating of working memory*** *The Colored Owls * A’—index	0.50 (0.26)	0.64 (0.23)	0.61 (0.21)	0.73 (0.12)	0.77 (0.10)	0.77 (0.13)	9.540	**0.000**
***Inhibition of automated responses*** *The Zoo Game * Commission Errors (%)	15.50 (13.17)	12.76 (10.82)	15.54 (13.01)	14.78 (12.78)	11.52 (9.80)	14.23 (12.06)	0.568	0.724
***Interference suppression*** *Catch the Frog * Interference Effect with RT (in ms)	30.45 (75.82)	9.27 (106.26)	38.88 (83.57)	23.39 (91.63)	30.52 (74.98)	31.50 (41.82)	0.458	0.807
Interference Effect with percentage of Errors	6.00 (14.99)	−0.36 (8.68)	0.41 (4.21)	−0.07 (7.93)	0.00 (3.32)	−0.46 (4.13)	2.342	**0.043**
***Set shifting*** *Find the Way * Task-Switching Cost with RT (in ms)	−211.21 (1095)	−22.36 (548.98)	186.38 (491.85)	131.18 (424.19)	138.00 (285.56)	26.17 (265.03)	1.616	0.158
Task-Switching Cost with percentage of Errors	0.29 (10.50)	6.36 (11.20)	14.88 (21.26)	7.98 (14.76)	5.09 (14.66)	7.38 (16.41)	2.386	**0.040**

**Table 6:** Means and standard deviations for each age group for hot EF tasks.

	Age Group							
Hot Executive Function	6 *n* = 20	7 *n* = 30	8 *n* = 37	9 *n* = 48	10 *n* = 34	11 *n* = 26	*F*	*p*
***Decision making*** *The Hungry Donkey * Net Score	3.30 (24.72)	9.27 (19.83)	2.49 (16.36)	6.96 (16.46)	3.27 (20.23)	0.31 (17.52)	0.942	0.455
***Delay of gratification*** *Planes and Rewards * Area under the curve	0.63 (0.32)	0.56 (0.24)	0.61 (0.27)	0.62 (0.30)	0.55 (0.25)	0.62 (0.25)	0.471	0.798
***Affective reversal learning*** *The Mechanic’s Game * Perseverance errors (%)	1.94 (2.75)	1.85 (2.14)	1.59 (1.76)	1.83 (1.25)	1.03 (1.38)	1.48 (1.56)	1.205	0.308
Regression errors (%)	4.28 (2.95)	7.44 (7.20)	6.16 (5.06)	5.19 (4.25)	5.44 (4.25)	4.32 (3.42)	0.928	0.464
Switching errors (%)	2.05 (1.61)	2.30 (1.96)	2.28 (1.75)	2.29 (2.33)	2.15 (1.44)	2.00 (1.22)	0.358	0.877
***Theory of mind*** *Playing with Jack * Accuracy (%)	22.50 (26.78)	30.83 (33.27)	43.24 (38.03)	44.79 (41.24)	41.91 (40.70)	58.65 (40.59)	2.614	**0.026**

**Table 7:** Means and standard deviations for the 11-year-old and adult groups for cool tasks.

	Age Group			
Cool Executive Function	11	Adults	*t*	*p*
***Updating of working memory*** *The Colored Owls * A’—index	0.77 (0.13)	0.89 (0.08)	−4.102	**0.000**
***Inhibition of automated responses*** *The Zoo Game * Commission errors (%)	14.23 (12.06)	10 (10.83)	1.383	0.172
***Interference suppression*** *Catch the Frog * Interference Effect with RT (in ms)	31.50 (41.82)	18.20 (37.99)	1.247	0.218
Interference Effect with percentage of Errors	−0.46 (4.13)	0.27 (2.77)	−0.784	0.436
***Set shifting*** *Find the Way * Task-Switching Cost with RT (in ms)	26.17 (265.03)	22.83 (154.58)	0.056	0.955
Task-Switching Cost with percentage of Errors	7.38 (16.41)	3.62 (9.90)	1.016	0.316

**Table 8:** Means and standard deviations for the 11-year-old and adult groups for hot tasks.

	Age Group			
Hot Executive Function	11	Adults	*t*	*p*
***Decision making*** *The Hungry Donkey * Net Score	0.31 (17.52)	11.67 (25.48)	−1.914	0.061
***Delay of gratification*** *Planes and Rewards * Area under the curve	0.62 (0.25)	0.68 (0.28)	−0.897	0.374
***Affective reversal learning*** *The Mechanic’s Game * Perseverance errors (%)	1.48 (1.56)	1.87 (2.01)	−0.784	0.436
Regression errors (%)	4.32 (3.42)	8 (9.06)	−2.06	**0.05**
Switching errors (%)	2.00 (1.22)	2.03 (1.65)	−0.084	0.934
***Theory of mind*** *Playing with Jack * Accuracy (%)	58.65 (40.59)	82.50 (31.59)	−2.426	**0.019**

## Data Availability

Data are contained within the article.

## Supplementary Materials

The following supporting information can be downloaded at: https://www.mdpi.com/article/10.3390/brainsci14080755/s1, Table S1: Previous executive batteries to assess executive functions in middle childhood [21,22,23,24,26,27,82].
