# Supplementary material for: A Novel Battery to Assess “Cool” and “Hot” Executive Functions: Sensitivity to Age Differences in Middle Childhood
Source: Brain Sci. 2024 Jul 27;14(8):755. doi: 10.3390/brainsci14080755 (PMC11352394; doi:10.3390/brainsci14080755)
Supplement: Supplementary file 1 [file brainsci-14-00755-s001.zip › brainsci-3072304-supplementary.pdf]

Table S1.

Previous executive batteries to assess executive functions in middle childhood.

Batteries	Cool EFs tasks	Hot EFs tasks	Ages (years)
**K-DESF** (Delis, Kaplan, & Kramer, 2001)	*Inhibitory control*: Color-Word Interference Test<br>*Cognitive flexibility:* Trail Making Test, Sorting Test, Word Context Test		8-89
**NIH Toolbox Cognition Battery** (Gershon et al., 2013)	*Inhibitory control*: Flanker inhibitory control, Attention Test<br>*Working memory*: List Sorting Working Memory Test<br>*Cognitive flexibility:* Dimensional Change Card Sort Test		3-85
**ENFEN** (Portellano et al., 2009)	*Inhibitory control, Working memory y Cognitive flexibility*: Trail Making Test<br>*Inhibitory control y Cognitive flexibility*: Stroop test		6-12
**ENI** (Matute, Rosselli, Ardila, & Ostrosky, 2007)	*Cognitive flexibility*: Clasificación de tarjetas		5-16
**CANTAB** (Fray, Robbins & Sahakian, 1996)	*Inhibitory control*: Stop Signal Task<br>*Working memory*: Spatial Working memory<br>*Cognitive flexibility*: Intra-Extra Dimensional Set Shift test, Multitasking Test	*Decision-making*: Gambridge Gambling Task<br>*Theory of mind*: Emotional Bias Task, Emotion Recognition Task	4-16
**BANFE** (Flóres, Ostrosky & Lozano, 2012	*Inhibitory control*: Stroop task,<br>*Working memory*: Señalamiento auto-dirigido, Ordenamiento alfabético de palabras, Memoria de trabajo viso-espacial secuencial	*Decision-making*: Juego de cartas	6-90

	Cognitive flexibility: Clasificación de cartas		
**NEPSY-II** (Korkman, Kirk & Kemp, 2007)	*Inhibitory control*: Inhibition task,<br>*Cognitive flexibility*: Animal Sorting task, Design Fluency task<br>*Control inhibitorio y Working memory*: Response Set task	*Theory of mind:* Affect Recognition task, Theory of Mind task	3-16
